# The Beneficial Effects of Quercetin, Curcumin, and Resveratrol in Obesity

**DOI:** 10.1155/2017/1459497

**Published:** 2017-08-24

**Authors:** Yueshui Zhao, Bo Chen, Jing Shen, Lin Wan, Yinxin Zhu, Tao Yi, Zhangang Xiao

**Affiliations:** ^1^Laboratory of Molecular Pharmacology, Department of Pharmacology, School of Pharmacy, Southwest Medical University, Luzhou, Sichuan, China; ^2^Experiment Center for Medical Science Research, Kunming Medical University, Kunming, Yunnan, China; ^3^Department of Hematology and Oncology, The Children's Hospital of Soochow, Jiangsu, China; ^4^Department of Gastroenterology, The Third Affiliated Hospital of Soochow University, Changzhou, Jiangsu, China; ^5^School of Chinese Medicine, Hong Kong Baptist University, Kowloon, Hong Kong; ^6^Key Laboratory of Medical Electrophysiology of Ministry of Education, School of Pharmacy, Southwest Medical University, Luzhou, Sichuan, China

## Abstract

Over the past two decades, obesity has been one of the major public health concerns in most countries. In the search for new molecules that could be used for the treatment of obesity, good perspectives have been opened up for polyphenols, a class of natural bioactive phytochemicals. Experimental and limited clinical trial evidence supports that some polyphenols such as quercetin, curcumin, and resveratrol have potential benefit functions on obesity treatment. This brief review focuses on the main functions of the above-named polyphenols on adipose tissue. These polyphenols may play beneficial effects on adipose tissue under obese condition by alleviating intracellular oxidative stress, reducing chronic low-grade inflammation, inhibiting adipogenesis and lipogenesis, and suppressing the differentiation of preadipocytes to mature adipocytes.

## 1. Introduction

Over the past decades, obesity has been one of the major public health threats in most developed countries and in an increasing number of developing countries [1]. Obesity is caused by the imbalance between energy intake and expenditure, which promotes the hypertrophy of adipocytes and results in adipose tissue dysfunction [2]. It is well known that obesity is a strong risk factor for type 2 diabetes mellitus (T2DM) and cancer, and T2DM is linked to the development of cardiovascular diseases, such as hypertension and atherosclerosis [3, 4]. Furthermore, obesity was associated with higher disability rates and mortality rates in the elderly [5]. A better understanding of the molecular basis of obesity will lead to establish strategies for prevention and treatment of obesity.

Adipose tissue is composed of many kinds of cell types, including adipocytes, macrophages, endothelial cells, and stem cells. In addition, as the major energy storage organ, adipose tissue also is a very important endocrine organ [6]. To maintain the function on energy regulation, adipose tissue produces adipokines, such as adiponectin and leptin, and proinflammatory cytokines, such as tumor necrosis factor- (TNF-) *α* and interleukin- (IL-) 1*β* [6]. Under normal physiological lean state, when the body takes excessive energy, adipose tissue can be rapidly enlarged by increasing the adipocyte size (hypertrophy) and numbers (hyperplasia), which were accompanied by an increase of blood vessels (angiogenesis) to supply more oxygen (O_2_) and nutrients to the whole tissue [7]. However, under pathological obese state, adipose tissue will undergo a process named “adipose tissue remodeling,” which was characterized by reduced angiogenesis, increased hypoxia levels and extra cellular matrix (ECM) levels, and induced higher levels of immune cell infiltration and subsequently induced a low-grade chronic inflammation. All of these pathological events will lead to adipocyte dysfunction, cell death, and systemic insulin resistance [7].

There are two types of adipose tissue, white adipose tissue and brown adipose tissue. The excess energy was mainly stored in the white adipose tissue in the form of triglycerides (TAGs). The function of brown adipose tissue is to directly transfer energy from nutrients to heat by uncoupling protein (UCP) 1, which mediates uncoupling of oxidative phosphorylation from ATP synthesis (conferred thermogenesis) [8–10]. *In vitro* and *in vivo* studies demonstrated that the activation of brown adipocytes is an effective and efficient way for excess energy metabolism [11–15]. Human studies showed that activation of brown-like adipocytes is a potential way to counteract obesity [12, 13, 16–19].

Oxidative stress is referred to an event resulting from the imbalance between the intracellular oxidation system and reduction system, the redox system [20]. The imbalance between oxidant and antioxidant enzymes/substrates will result in a series of oxidation-reduction reactions, which will subsequently induce cytotoxicity by inducing cellular stress responses and stimulating cell death [21]. A series of studies have revealed that oxidative stress is related to the development of obesity. Excess levels of reactive oxygen species (ROS) might lead to the dysfunction of mitochondria by inhibiting respiration process and result in a reduction on the energy expenditure in adipocytes and conversely enhance the energy storage in adipose tissue [22]. Oxidative stress also suppresses the endocrine functions of adipose tissue by disrupting the secretion of adipokines such as adiponectin [23]. Antioxidants can protect cells from oxidative stress by trapping free radicals and restoring cell functions. In recent years, chemical antioxidants derived from natural plants, which are named as “phytochemicals,” have gained interest by researchers for preventing and treating diseases, including obesity and obesity-related metabolic diseases [24–29].

Among the phytochemicals studied, researchers pay more attention on polyphenols, which are derived from diet food such as vegetables and fruits, as well as beverages such as juice, coffee, and tea [30–35]. Studies showed that polyphenols such as quercetin, curcumin, and resveratrol exerted beneficial effects on lipid and energy metabolism and potential body weight change. In this review, we will focus on the roles of and the mechanisms of polyphenols including quercetin, curcumin, and resveratrol and on obesity and adipose tissue function.

## 2. Quercetin

Quercetin is the most abundant of flavonoids and is found in vegetables, fruits, tea, and wine [36].

### 2.1. Effects on Cell Culture Models of Obesity

The first *in vitro* study investigating the potential antiobesity effect of quercetin on obesity was performed on primary adipocytes. Kuppusamy and Das found that quercetin induced lipolysis of primary rat adipocytes in a dose- and time-dependent manner by increasing cyclic adenosine monophosphate (cAMP) levels and hormone-sensitive lipase (HSL) activity [37]. In addition to the inductive effect on lipolysis, quercetin can also suppress lipogenesis by reducing the incorporation rate of fatty acids into adipocyte triacylglycerols in rat fat pads [38] and by inhibiting the gene expression levels of fatty acid synthase (FAS) and the activity of acetyl-CoA carboxylase (ACC) [39]. Quercetin also can inhibit adipogenesis by decreasing gene expression levels of the key adipogenic factors peroxisome proliferator-activated receptor *γ* (PPAR*γ*) and CCAAT/enhancer binding protein *α* (C/EBP *α*) [39]. Recently, using hypertrophied 3T3-L1 adipocyte model, Herranz-López et al. showed that quercetin can rapidly reduce the intracellular ROS levels, which was correlated with the higher levels of quercetin metabolite [40]. Moreover, in human SGBS adipocytes, quercetin can significantly reduce levels of adipokines ANGPTL4, adipsin, and PAI-1 as well as of glycolysis-associated enzymes ENO2, PFKP, and PFKFB4, all of which are associated with obesity and adipose tissue dysfunction [41]. Adipocyte browning is a promising strategy for the prevention of obesity [14, 42–44]. In 3T3-L1 adipocytes, quercetin (50 *μ*M) induced the expression of brown adipocyte-specific genes such as UCP-1 and cell death-inducing DNA fragmentation factor-alpha-like effector A (CIDEA) by the activation of AMP-activated protein kinase (AMPK) [45], which is a key checkpoint to control the energy balance in adipocytes by suppressing the activity of ACC; as a result, the levels of lipid in adipocytes were decreased [46].

### 2.2. Effects on Animal Models of Obesity

Animal studies showed that quercetin can protect mice or rats from high-fat diet- (HFD-) induced body weight gain and adipose tissue accumulation [47–49]. In HFD-fed mouse model, Stewart et al. showed that quercetin can transiently increase energy expenditures which may relate to the upregulation of UCP-1 [49]. In HFD-fed rat model, quercetin suppressed adipogenesis by reducing the key adipogenic factor C/EBP *α* gene expression levels and reduced lipogenesis by downregulating the gene levels of FAS and ACC [50]. Quercetin also has anti-inflammatory effects on adipose tissue. Stewart et al. found that long-time treatment with quercetin can reduce the levels of inflammatory markers IFN*γ*, TNF*α*, IL-1, and IL-4 in mice [49]. Quercetin suppresses the accumulation and activation of immune cell and improves mitochondrial functions in adipose tissue of HFD-induced obese mice by increasing the levels of oxidative stress-sensitive transcription factor and antioxidant enzymes [51]. Moreover, Dong et al. found that quercetin attenuated mast cell and macrophage infiltration into epididymis adipose tissues (EATs) through the AMPK *α*1-silent information regulator (SIRT) 1 pathway in HFD-fed mice [52]. In Wistar rats, quercetin suppressed the expression of oxidative stress and inflammatory markers, including nuclear factor kappa B (NF-*κ*B), nuclear factor-related factor- (Nrf-) 2, and heme oxygenase- (HO-) 1 [53]. In another study, quercetin (10 mg/kg of body weight) improved the inflammatory status of visceral adipose tissue by suppressing the expression of TNF-*α* and enhancing the levels of adiponectin, which indicates the recovery of the functions of the adipose tissue, in obese Zucker rats, a genetically obese rat model [48].

### 2.3. Human Studies and Clinical Trials Using Quercetin to Treat Obesity

Although many cell culture and animal studies focused on the beneficial effects of quercetin in obesity, there are only a limited number of human studies and clinical trials that have been performed to evaluate the effects of quercetin on obesity treatment. In a 12-week, randomized, double-blind, placebo-controlled study, Lee et al. demonstrated that quercetin (100 mg/day/subject) significantly decreased the total body fat, particularly in the percentage of fat in the arm, and decreased the body mass index (BMI) of overweight or obese subjects [54]. Another study evaluated the effects of quercetin on obesity in overweight-obese subjects with various apolipoprotein E (APOE) genotypes; the authors reported that quercetin (150 mg/day/subject) decreased the waist circumference and triacylglycerol concentration [55]. In addition to these findings, one study showed that 12-week of onion extract (quercetin-rich extract) intake decreased body weight, percentage of body fat, and BMI of 10 female university students [56]. However, another study reported that 12-week of onion extract intake has no effect on body fat composition and BMI of the female university students [57], indicating that the experiment period of the study is important for the effects of the onion extracts on body weight change. Currently, there is one clinical trial that is still under phase II stage investigation; the purpose of this study is to investigate whether quercetin changes the absorption of glucose by the body in obese subjects and obese diabetic subjects [58]. Although quercetin suppressed oxidative stress in obese rodent models [51, 53], Shanely et al. reported that quercetin has no effect on oxidative stress and antioxidant capacity during a 12-week consuming period of high doses of quercetin (500 or 1000 mg/day/subject) in obese subjects [59]. Future research need to further investigate the bioactive effects and bioavailability of quercetin in the treatment of obesity.

## 3. Curcumin

Curcumin is derived from and is the most bioactive polyphenol in the spice turmeric [60]. Curcumin exerts several biological functions including antioxidation, anti-inflammation, and antiangiogenesis in different organs including adipose tissue [60].

### 3.1. Effects on Cell Culture Models of Obesity

Curcumin may have a significant effect on adipogenesis. In primary human adipocytes and murine 3T3-L1 adipocytes, curcumin treatment suppressed the expression of adipogenic genes peroxisome proliferator-activated receptor *γ* (PPAR*γ*) and C/EBP *α* [61]. In addition to the antiadipogenic effects, curcumin also suppresses the differentiation of preadipocytes to mature adipocytes. Ahn et al. demonstrated that curcumin inhibited 3T3-L1 adipocyte differentiation by inhibiting activities of mitogen-activated protein kinases including ERK, JNK, and p38 [62]. Another report showed that the inhibition effect of curcumin on adipocyte differentiation might have been mediated by the suppression of PPAR*γ* expression in a dose-dependent manner in human adipocytes [63]. Moreover, curcumin also showed anti-inflammatory effects. Curcumin pretreatment inhibited the secretion of monocyte chemoattractant protein- (MCP-) 1, a proinflammatory cytokine, from 3T3-L1 adipocytes [64].

### 3.2. Effects on Animal Models of Obesity

Curcumin showed beneficial effects on body weight reduction and energy metabolism. Two weeks of high dietary curcumin supplementation feeding in rats reduced epididymal adipose tissue and increased fatty acid *β*-oxidation, indicating the increase of energy expenditure after curcumin treatment [65]. Curcumin also showed anti-inflammatory functions. In HFD-induced obesity and in genetic obesity (ob/ob mice) models, curcumin reduced adipose tissue inflammation by reducing macrophage infiltration into adipose tissue and by increasing adiponectin production [66, 67]. Curcumin also showed antioxidant effects. Dietary curcumin (0.2–1 g/100 g diet) suppressed high-fat-induced lipid accumulation in epididymal adipose tissue [65].

### 3.3. Human Studies and Clinical Trials Using Curcumin to Treat Obesity

Unlike the studies on the effects of curcumin in cells or animals, studies on obese subjects are limited. The first clinical trial using curcumin for obesity treatment was conducted by Mohammadi et al. [68]. In this study, obese subjects were treated with a commercial formulation of curcumin (C3 Complex^®^, 1 g/day) supplemented with a bioavailability enhancer, piperine (5 mg/day) for a month. Although there were no changes in weight, body mass index (BMI), or body fat, serum triglyceride levels were significantly decreased after curcumin treatment, indicating the improvement of insulin actions [68]. In another randomized, double-blind, crossover trial, Ganjali and Sahebkar showed that 30-day treatment of C3 Complex (500 mg/day) plus piperine (5 mg/day) reduced serum levels of inflammatory cytokines IL-1*β* and IL-4 of obese individuals [69], indicating the anti-inflammatory activity of curcumin in obesity therapy. Moreover, oral curcumin supplementation (1 g/day for 30 days) was effective in reducing oxidative stress burden in obese individuals [70].

Although curcumin has been used for clinical trials in obesity treatment, the multifaceted pharmacological nature of curcumin and its pharmacokinetics and the side effects of curcumin in obesity therapy need to be carefully investigated. The recommended maximum daily usage of curcumin is 1 mg/kg body weight by a joint report of the World Health Organization and the Food and Agriculture Organization [71]. However, a few studies showed that the chronic use of curcumin can cause liver toxicity [72] and high doses of curcumin can induce gastrointestinal upset, inflamed skin, and chest tightness in a phase II trial in patients with advanced pancreatic cancer [73].

## 4. Resveratrol

Resveratrol (3,5,4′-trihydroxytrans-stilbene) is a small polyphenolic compound, which was well known as constituent of red grapes, red wine, peanuts, and ground nuts [74, 75]. Resveratrol showed antioxidant and anti-inflammatory actions [76] and showed beneficial effects in preventing the development of many diseases including obesity and diabetes [77].

### 4.1. Effects on Cell Culture and Ex Vivo Adipose Tissue Culture Models of Obesity

Resveratrol can inhibit adipogenesis by reducing the stability and transcriptional activity of PPAR*γ* [78, 79] and prevent triglyceride accumulation via enhancing the expression of sirtuin1 (Sirt1), which is an important molecular target regulating cellular energy metabolism and mitochondrial homeostasis [80] in 3T3-L1 adipocytes. Moreover, resveratrol enhanced lipolytic activity in human and rat adipocytes; this effect was mediated by *β*-adrenergic activation and the induction of cAMP levels [81, 82]. In addition, to enhance lipolysis, resveratrol also can inhibit lipogenesis by downregulating the expression of lipogenic genes in human adipocytes [83]. Kang et al. found that resveratrol pretreatment suppressed secretion of TNF-*α* and IL-6 from 3T3-L1 adipocytes and inhibited the activation of inflammatory-related proteins such as extracellular receptor-activated kinase (ERK) and NF-kappaB (NF-*κ*B), indicating that resveratrol has anti-inflammatory effects in adipocytes [84]. In human adipocytes, resveratrol reversed IL-1*β*-stimulated expression of proinflammatory adipokines including IL-6, IL-8, monocyte chemoattractant protein- (MCP-) 1, and plasminogen activator inhibitor- (PAI-) 1 [85, 86]. Moreover, reports showed that resveratrol inhibited adipose tissue inflammation by downregulating the protein levels of IL-6, IL-8, MCP-1, and the inflammatory-related adipokine leptin in human adipose tissue *in vitro* [86, 87].

### 4.2. Effects on Animal Models of Obesity

Dietary treatment of rodents with resveratrol protected mice against HFD-induced body weight gain and obesity by increasing energy expenditure which was partly mediated by stimulating intracellular mitochondrial functions (fatty acid oxidation) in adipose tissue and by the suppression of fatty acid synthesis [88–90] and by inducing brown-like adipocyte formation in white adipose [91–94]. The *in vitro* anti-inflammatory effect of resveratrol was also confirmed in animal models. In mice, resveratrol attenuated HFD-induced inflammation of WAT by downregulating the protein levels of proinflammatory cytokines TNF-*α*, IFN-*α*, IFN-*β*, and IL-6 [89]. In addition, resveratrol reduced adipose tissue macrophage infiltration [95] and prevented the suppression of the production of regulatory T cells (Tregs, the negative regulators of inflammation) [96] in HFD-induced obese mice. In Zucker rats, resveratrol suppressed the protein levels of IL-6 and the activity of NF-*κ*B in adipose tissue by reducing macrophage infiltration [97]. Interestingly, Jimenez-Gomez et al. showed that resveratrol showed similar effects on high-fat-treated adult rhesus monkey model as effects on HFD-induced obese rodent models, suppressed the activation of NF-*κ*B, and decreased the mRNA levels of IL-6, TNF-*α*, IL-1*β*, and adiponectin in the visceral adipose tissue of high-fat-treated monkey model [98]. Resveratrol also showed antioxidant effect in animal models. Lv et al. found that resveratrol attenuated diet-induced oxidative stress in epididymal white adipose tissue partly by the reduction of Sirt1 and manganese superoxide dismutase (Sod2) levels [99].

### 4.3. Clinical Trials Using Resveratrol to Treat Obesity

Although several clinical trials that examine the effect of resveratrol on obesity are currently ongoing (see http://clinicaltrials.gov) or have finished (see Review [100]), none of them were designed specifically to test the effects of resveratrol on body weight change of obese subjects. In a randomized double-blind cross-over study, Timmers et al. showed that 150 mg/day of resveratrol treatment increased energy expenditure, reduced serum inflammatory markers, and decreased adipose tissue lipolysis and plasma fatty acid and glycerol levels of obese men [101]. In another study, Konings et al. investigated the effects of 30 days resveratrol treatment (150 mg/day) on the adipocyte size and gene expression patterns in obese men. The authors found that resveratrol treatment decreased the size of abdominal subcutaneous adipocytes [102]. However, another report showed that high levels of resveratrol supplementation treatment had no effect on energy expenditure, adipose tissue content, and metabolic events [103]. The reason for the reversed results obtained from the two reports may possibly lie in the administered doses of resveratrol they used for obesity treatment. The latter report used 1500 mg/day for the trial [103]; this dose was ten times of the dose Konings et al. used in the study [102].

## 5. Concluding Remarks

In the search for new molecules that could be used for the treatment of obesity, good perspectives have been opened up for polyphenols. Current knowledge from cell cultures and animal models suggests that polyphenols, including quercertin, curcumin, and resveratrol, play beneficial effects under obese condition potentially by alleviating intracellular oxidative stress, reducing chronic low-grade inflammation, inhibiting adipogenesis and lipogenesis, and suppressing the differentiation of preadipocytes to mature adipocytes. Although investigators have obtained limited results from clinical trials, there is still no sufficient data to support the high-dose and long-term usage of these polyphenols in obesity treatment.
